# Sidenafil Pre-Treatment Promotes Decompression Sickness in Rats

**DOI:** 10.1371/journal.pone.0060639

**Published:** 2013-04-08

**Authors:** Jean-Eric Blatteau, Alf O. Brubakk, Emmanuel Gempp, Olivier Castagna, Jean-Jacques Risso, Nicolas Vallée

**Affiliations:** 1 Equipe Résidante de Recherche Subaquatique Opérationnelle, Département Environnements Opérationnels/Environnements Extrêmes, Institut de Recherche Biomédicale des Armées, Toulon, France; 2 Department of Physiology and Biomedical Engineering, Faculty of Medicine, Medical Technology Center, Norwegian University of Science and Technology, Trondheim, Norway; 3 Laboratoire Motricité Humaine, Education Sport et Santé, Equipe d’Accueil 6309, Université de Toulon, Toulon, France; 4 Service de Médecine Hyperbare et Expertise Plongée, HIA Sainte-Anne, Toulon, France; Hôpital Robert Debré, France

## Abstract

Vascular bubble formation after decompression contributes to endothelial injuries which form the basis for the development of decompression sickness (DCS). Nitric oxide (NO) is a powerful vasodilator that contributes to vessel homeostasis. It has been shown that NO-releasing agent may reduce bubble formation and prevent serious decompression sickness. The use of sildenafil, a well-known, phosphodiesterase-5 blocker, which act by potentiating the vasodilatory effect on smooth muscle relaxation, has never been studied in DCS. The purpose of the present study was to evaluate the clinical effects of sildenafil pre-treatment on DCS in a rat model. 67 rats were subjected to a simulated dive at 90 msw for 45 min before staged decompression. The experimental group received 10 mg/kg of sildenafil one hour before exposure (n = 35) while controls were not treated (n = 32). Clinical assessment took place over a period of 30 min after surfacing. At the end, blood samples were collected for blood cells counts and the level of circulating bubbles in the right cavities was quantified. There were significantly more manifestations of DCS in the sildenafil group than in the controls (34.3% vs 6.25%, respectively, p = 0.012). Platelet count was more reduced in treated rats than in controls (−21.7% vs −7%, respectively, p = 0.029), whereas bubble grades did not differ between groups. We concluded that pre-treatment with sildenafil promotes the onset and severity of neurological DCS. When considering the use of phosphodiesterase-5 blockers in the context of diving, careful discussion with physician should be recommended.

## Introduction

Scuba (self contained underwater breathing apparatus) diving is an activity growing in popularity every year. Today the diving population is becoming older and includes many individuals who are relatively unfit, taking medication. Scuba diving may result in the production of bubbles due to the release of inert gas originally held in solution in the form of a free gas phase from peripheral tissues during decompression. When bubbles are excessively generated in blood and tissues, signs and symptoms referred to as decompression sickness (DCS) may occur [1]. It is generally accepted that gas bubbles grow from pre-formed gas nuclei attached to the vessel walls [2] and that venous gas emboli magnitude is linked to an increased risk of DCS [3], [4]. Neurological damage in the spinal cord and brain underlies the most serious symptoms of DCS [5]. Even after standard treatment with hyperbaric oxygen, 20–30% of the divers affected by neurological DCS had incomplete recovery at discharge [6]. It is acknowledged that vascular bubble formation contribute to endothelial injury leading to vascular obstruction, microcirculatory alterations and activation of coagulation cascades, which may form the basis for the development of DCS [5], [7].

It has been shown that endothelial nitric oxide (NO) is a critical factor in the development of bubble formation and decompression sickness [8]. NO, which is gas highly soluble in lipids, may cross biological membranes and diffuse to the subjacent vascular smooth muscle where it regulates vascular tones by activation of the enzyme guanylate cyclase. This increases intracellular cyclic guanosine monophosphate (cGMP) concentrations leading to vasodilation from relaxation of the smooth muscle cells. NO may also act on the luminal endothelial surface by inhibiting platelet aggregation and leukocyte adhesion, scavenging free radicals and modulating the permeability of endothelial layer [9], [10]. NO, through its multiple properties, could facilitate the elimination of vascular gaseous nuclei, thereby reducing the formation of bubbles during decompression. Previous studies have shown that blockade of NO synthesis with L-NAME dramatically increases peripheral vascular resistances and bubble formation in rats with a reduction of survival after decompression [11]. Conversely, prolonged or immediate (30 minutes) administration of NO-releasing agent (isosorbid mononitrate) before simulated diving protects animals against bubble formation and death [8], [12]. Similar results were obtained on volunteer human divers by demonstrating that application of short-acting NO donor (0.4 mg of nitroglycerine by oral spray) 30 minutes before the dive reduced the number of venous circulating bubbles [13].

However, the use of phosphodiesterase (PDE)-5 blockers, which act by potentiating the action of cGMP in vascular smooth muscle cells, has never been studied in DCS. Sildenafil, sold as Viagra™, Revatio™, and under various other trade names, is a selective and potent inhibitor of PDE type 5 which specifically degrades cGMP and is found in platelets, skeletal muscle, and visceral and pulmonary vascular smooth muscle, as well as in cerebral neurons and vessels [14], [15]. However the highest concentrations are observed in pulmonary arteries and the corpora cavernosum [16], [17]. For a decade, sildenafil citrate has become one of the top-selling products in the world-wide market for male impotence treatment. Inhibition of PDE-5 present in the endothelium of the corpora cavernosa prolongs the relaxation of the pericavernous smooth muscle, sustaining its engorgement with blood [18]. Sildenafil is generally well tolerated, with most side effects related to vasodilation i.e. headache, flushing [19], [20]. However, recent concern and media coverage of temporally related cardiovascular events, including myocardial infarction, arrhythmias, and death, reported after the release of sildenafil onto the market raised questions regarding the safety of sildenafil in patients with cardiovascular disease [21]. Other serious but rare adverse effects have been also reported, i.e. life-threatening intracerebral hemorrhage in a young adult male [22]. Indeed, sildenafil may act by redistributing arterial cerebral blood flow, which may result in some cases to rupture of vessels. PDE-5 inhibitors may also increase the risk of central nervous system (CNS) oxygen toxicity by enhancing cerebral blood flow under conditions that can occur in diving or during hyperbaric oxygen administration [23].

Based on its properties, sildenafil could have a beneficial role on decompression by reducing bubble formation. On the other hand, its vascular impact on the central nervous system is not known in the context of diving and severe decompression. To our knowledge, there are no reported data on the occurrence of DCS in divers taking sildenafil. The purpose of the present study was to evaluate the clinical effects of sildenafil pre-treatment on DCS in a rat model. To complete this analysis, we also determined the level of circulating vascular bubbles in the right cavities and counted blood cells.

## Materials and Methods

### Study Population

All procedures involving experimental animals were in line with European Union rules (Directive 86/609) and French law (Decree 87/848). The ethics committee of Institut de Recherche Biomédicale des Armées approved this study. Our investigator (NV) is associated to the agreement number 83.6 delivered by the Health and Safety Directorate of our department, as stated in the French rules R.214-93, R-214-99 and R.214-102.

Only male Sprague-Dawley rats (Harlan Laboratories, France) were used in this experiment in order to avoid fluctuations due to female hormone cycles. Rats were kept at 22±1°C in a 12∶00/12∶00-h light/dark cycle (lights on at 7∶00 a.m.) with food (A03, UAR) and water ad libidum. Before experiments, rats were housed in an accredited animal care facility. All procedures involving experimental animals were in line with European Union rules (Directive 86/609) and French law (Decree 87/848).

**Figure 1:**
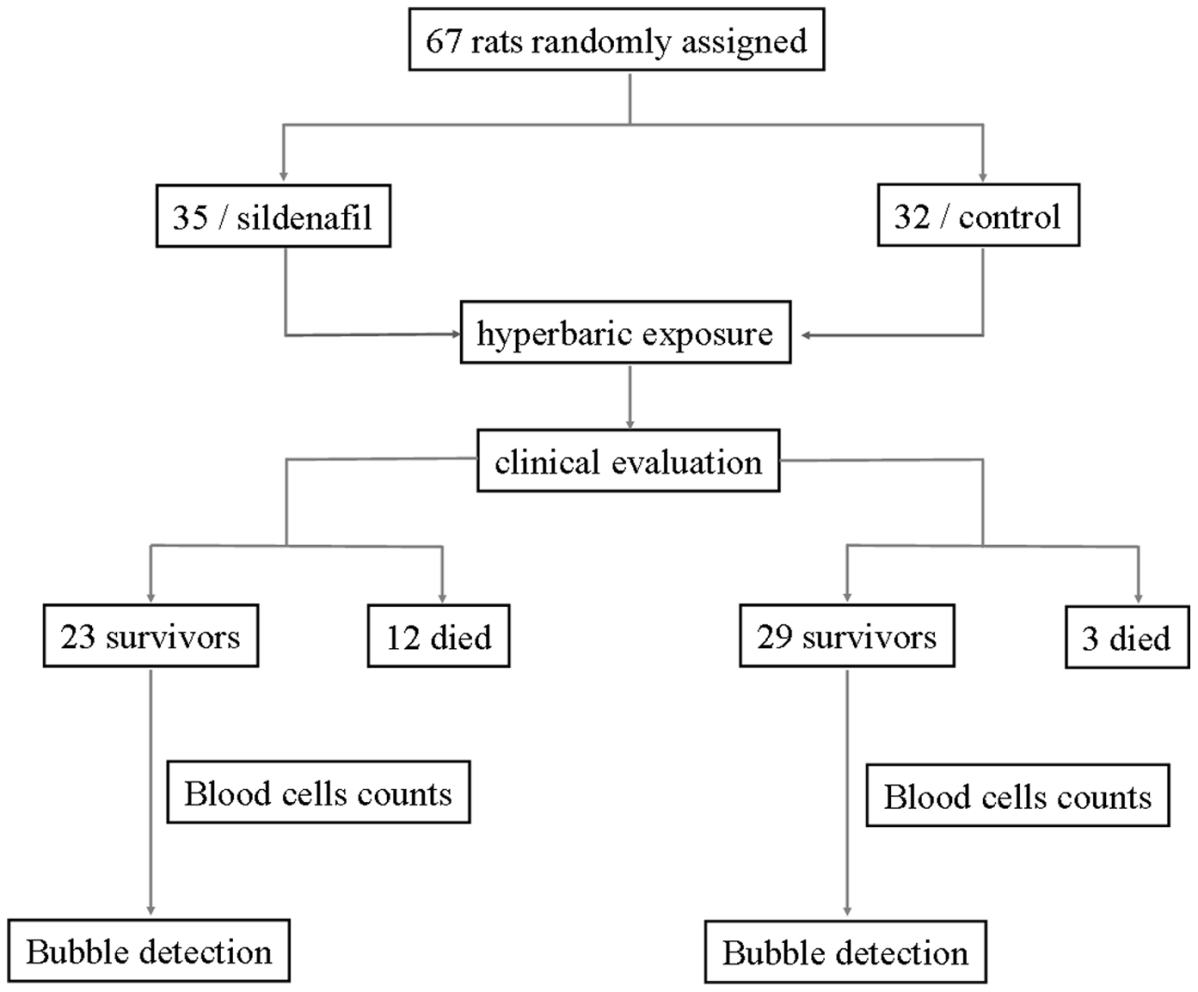
Flow chart describing the experimental design.

A total of 67 rats were exposed to compressed air to induce decompression stress and bubble formation. The rats were randomly divided into two groups and numbered: 35 for the group treated with sildenafil and 32 for the controls. The experimental design is detailed in Figure 1. Previous studies showed that bubble formation and incidence of decompression sickness were highly dependent upon body weight of the rat [24], [25], [26]. Protocols using rats with a body weight above 350 g produced severe DCS with severe neurological symptoms and death. Weight was similar in both groups (361±15 g for sildenafil vs 358±12 g for controls, p = 0.510). The rats of experimental group received a single gavage dose of 10 mg/kg of sildenafil solution (0.5 ml) in the form of Viagra™ (Pfizer) 1 hour before hyperbaric exposition while the control group received a similar volume of water (0.5 ml) without sildenafil. The selected dose has been previously shown to be effective in a rat model of embolic stroke [27] and time of administration of sildenafil was adjusted according its onset of action with a peak at 60 minutes.

### Hyperbaric Procedure

Batches of 10 freely-moving rats (8–10 per cage) were subjected to the hyperbaric protocol in a 200-liter tank fitted with three ports for observation.

Rats underwent the compression procedure at a rate of 100 kPa.min^−1^ to a pressure of 1000 kPa (90 msw), maintained for 45 min while breathing air. At the end of the exposure period rats were decompressed down to 200 kPa at a rate of 100 kPa.min^−1^ with a 5-min stop at 200 kPa, a 5-min stop at 160 kPa, and a 10-min stop at 130 kPa. Decompression between 200 kPa and surface was performed at a rate of 10 kPa.min^−1^. The decompression rate was automatically controlled by a computer linked to an Analogical/Digital converter (NIUSB-6211, National Instrument, USA) itself connected to a solenoid valve (Belino LR24A-SR, Switzerland) and a pressure transmitter (Pressure Transmitter 8314, Burket Fluid Control System, Germany). The program used to control decompression rate was designed on DasyLab (DasyLab National Instrument, USA) by our engineer.

Compressed air was generated using a diving compressor (Mini Verticus III, Bauer Comp, Germany) coupled to a 100-liter tank at 300 bars. The oxygen analyzer was based on a MicroFuel electrochemical cell (G18007 Teledyne Electronic Technologies/Analytical Instruments, USA).Water vapor and CO_2_ produced by the animals were respectively captured with seccagel (relative humidity: 40–60%) and soda lime (<300 ppm captured by the soda lime), respectively. Gases were mixed by an electric fan. The day-night cycle was respected throughout. The temperature inside the tank was measured using a platinum-resistance temperature probe (Pt 100, Eurotherm, France). All these variables were controlled by a dedicated computer.

### Behavior and Clinical Observations

At the end of decompression, the rats were transferred to individual cages and observed during 30 minutes by a dedicated staff, blinded to treatment. The following symptoms were considered as manifestations of DCS: labored breathing, paralysis or moving difficulties (including limping, failure to maintain balance, sideways gait, falling, difficulty righting after a fall), and death. The time of onset of these symptoms were also recorded. Problems with fore or rear limbs were classified as being due to neurological DCS.

### Blood Cells Tests

Blood tests were carried out in an automatic analyzer (ABCvet, SCIL, France) on samples taken before the dive and then again 30 minutes after surfacing. Red cells, leukocytes and platelets were counted in 20 µl samples taken from the tip of the tail and diluted in an equivalent volume of 2 mM EDTA (Sigma, France).

### Bubble Detection

The rats were anesthetized 30 minutes after surfacing by intraperitoneal injection of a mixture of 10 mg/kg xylazine (Rompum® 2%, Bayer Pharma) and 100 mg/kg ketamine (Imalgene®1000, Laboratoire Rhône, France). Bubble detection was performed using a Micromaxx Sonosite, with a probe of 4–8 Mhz. Bubbles were identified as bright spots in the right ventricule with 2D mode. Bubbles were graded according to the Spencer scale [4] using the pulsed Doppler mode. The observer was blinded to the group allocation of the rat. At the end of the experiment, the rats were sacrificed by injecting pentobarbital (200 mg/kg ip, Sanofi Santé, France).

### Statistical Analyses

For statistical processing, we used Sigmastat 3.0 (SPSS inc., Chicago, Illinois). Numerical results were expressed as median and interquartile range. A contingency table was used for independence and association tests, coupled with a Fisher Exact or Chi^2^ test of significance. Differences between two groups were analysed by a Mann-Whitney test, whereas matched comparisons within groups used a Wilcoxon test. A difference was considered as significant for p-values <0.05.

## Results

### Clinical Observations (Figure 2)

The rats expressed mainly neurological symptoms of varying intensity including locomotor impairment i.e. paraplegia or paraparesis, often followed by the death of the animal. All cases of neurological symptoms occurred within 5 min after surfacing. In a few cases, respiratory symptoms were also noted (2 cases in each group). There was significantly more neurological symptoms of DCS in the sildenafil group compared with the controls (34.3% vs 6.25%, respectively, p = 0.012). Death, generally preceded by paraplegia, occurred more frequently in the sildenafil group, with a significant difference (34.3% vs 9.4%, respectively, p = 0.032). Additional analysis found that time to death (9±13.5 min for sildenafil vs 1±6.5 min for controls, p = 0.515) and time to onset of DCS symptoms were not significantly different between groups (median of 1 min in each group, p = 0.882).

**Figure 2:**
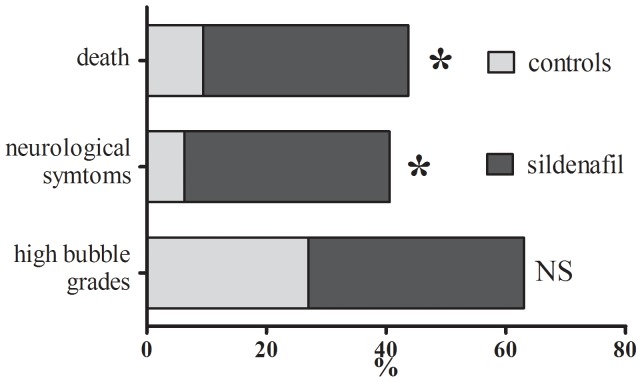
Percents of dead rats and animals presenting neurological symptoms of decompression sickness within 30 min after surfacing. Percents of rats with high bubble grades (Spencer grades 3 and 4) during precordial bubble detection are also represented. Histogram in dark grey represents the group treated with sildenafil and light grey represents the controls. * denotes p<0.05 between groups.

### Bubble Detection (Figure 2)

Bubbles were observed in 72.7% of animals treated with sildenafil and in 56.6% for controls, but this difference was not significant (p = 0.371). Moreover, no differences in bubble grades were observed between groups (p = 0.266), with a median grade of 2±3 for the sildenafil group and 1±3 for controls. High bubble grades i.e. Spencer grades 3 and 4 were found in 36% and 27% of treated animals and controls, respectively.

### Blood Cells (Figure 3)

#### Platelet counts

Following the dive, the platelet count was reduced by −21.7±25.4% from baseline in the sildenafil group while the decrease was only −7±13.8% in the controls. This difference between groups was significant (p = 0.029).

**Figure 3:**
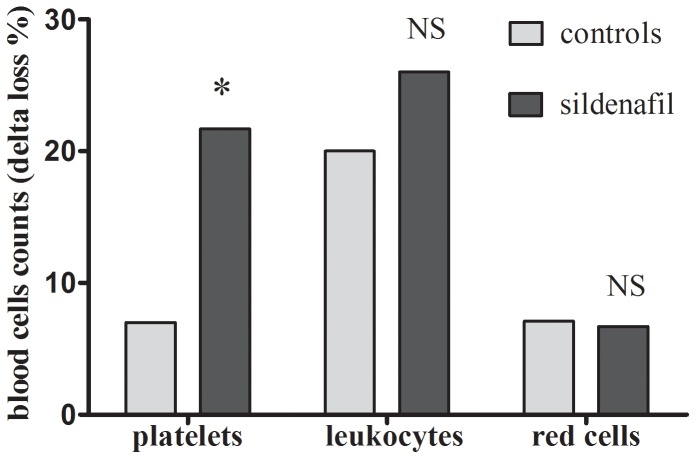
Percents of blood cells consumption after decompression from the baseline in dark grey for the group of rats treated with sildenafil and light grey for the controls. * denotes p<0.05 between groups.

#### Leukocyte counts

Following the dive, the leukocyte count was decreased from baseline by −26±53% in the sildenafil group and by −20±37% in the controls, with no statistical differences between groups (p = 0.739).

#### Red cells

Following the dive, the red cell went down by −6.7±10.3% from baseline in the sildenafil group and by −7.1±7.5% in the control group, with no statistical differences between groups (p = 0.431).

## Discussion

The aim of the present study was to investigate the effects of sildenafil in a clinically relevant model of DCS that produces motor impairment suggestive of neurological DCS [24], [25], [26]. The main finding in this study is that rats treated with sildenafil had greater DCS incidence, as assessed both by clinical observations and biomarkers such as platelets consumption.

We found that platelet count was more reduced in treated rats than in controls. This result reflects the severity of clinical DCS in the group treated with sildenafil. Indeed animal experiments strongly suggest a role for the involvement of blood components in DCS [5], [28]. Previous animal studies reported that platelet count falls following decompression and can be considered to be a relevant index for evaluating decompression stress [26], [28]. The drop in platelet count is usually attributed to clotting activity following exposure of the collagen under bubble-damaged endothelial cells in the blood vessels [7], [29], or direct interaction between bubbles and platelets [30], [31]. Concerning leukocytes and red cells, we did not find significant differences between groups. Experimental observations in DCS suggest that damage to the vascular endothelium by gas bubbles may provoke an inflammatory and immune response resulting in leukocyte activation. The fall in leukocyte count after DCS is usually attributed to diapedesis [32]. Several authors have observed phenomena of blood sludging and red-cell fragmentation/deformation following rapid decompression in animal models. The formation of red-cell aggregates appears to be associated with flow stasis [28]. However the use of leukocyte and red cell counts do not seem as powerful as platelet count in DCS evaluation [28].

We believe that the promoting effect of sildenafil on the occurrence of DCS may be related to vasodilation in the CNS with increased cerebral blood flow [23]. This effect may contribute to significantly increasing the load of inert gas during the hyperbaric exposure and thereby generate bubbling and severe DCS in neurological tissue. The elevation of cerebral blood flow can also be deleterious in specific circumstances. Demchenko et al. [23] demonstrated that conscious rats treated with sildenafil before an exposure to hyperbaric oxygen were more susceptible to CNS oxygen toxicity, as demonstrated by significantly shortened convulsive latency. This result demonstrated that sildenafil opposes the protective vasoconstriction that is the initial response to hyperbaric hyperoxia, by hastening onset of CNS oxygen toxicity. Since the neurological signs that we observed were primarily motor impairment with no case of convulsions, it is unlikely that relative hyperoxia (equivalent exposure to 90 msw in air atmosphere, 200 kPa O_2_) may have played a role in the onset of neurological symptoms in the present study. However it is possible that increased oxidative stress may have contributed to promote the onset of neurological DCS. Indeed, increased cerebral blood flow leading to increased oxidative stress may produce peroxynitrites, and several studies have reported such toxicity after ischemia and reperfusion [33] or traumatic brain injury [34]. However, in other circumstances the elevation of cerebral blood flow can be beneficial. Indeed, previous studies demonstrated that treatment of experimental stroke with sildenafil, initiated at 24 h after onset of ischemic stroke was associated with improvement of functional recovery compared with saline-treated rats [27], [35]. Cerebral blood flow values of the ischemic tissue along the ischemic lesion boundary area measured by MRI were significantly elevated in sildenafil treated rats compared with control rats [35]. These aforementioned experimental studies suggest that sildenafil could be useful as an adjuvant treatment of ischemic neurological DCS that have not recovered after initial treatment with hyperbaric oxygen.

Previous experimental studies have shown that NO donors i.e. nitrates are able to remove vascular bubbles both in rats and man [8], [12], [13], probably by eliminating gas nuclei before hyperbaric exposure. However, we found in our study, that sildenafil was not able to reduce bubble formation. Thus, NO donors must involve properties and mechanisms different from those encountered with sildenafil. This suggests that the presence of gas nuclei attached to the vessel wall is not directly influenced by the vasodilator effect related to the relaxation of the smooth muscle induced by the increase of cGMP. Indeed, sildenafil prevents the degradation of cGMP by inhibiting PDE-5, and therefore acts on cGMP by a mechanism different from the NO pathway. Apart from its vasodilator effect related to vascular smooth muscle relaxation, NO also diffuses to the luminal surface of the endothelium, where it exerts a number of important physiological effects, including scavenging of superoxide radicals, inhibition of platelet adherence and aggregation, modulation of endothelial layer permeability and attenuation of leukocyte function. We believe that these specific effects of NO, which are not reported with sildenafil, could be involved in the reduction of the number of gas nuclei adhering to the surface.

Our results do not render obsolete the previous works on the prevention of decompression sickness by preconditioning methods such as exercise or sauna that stimulate the production of endogenous NO and reduce bubble formation [36], [37]. Exogenous NO pre-treatment from low doses of nitrates may also reduce vascular bubbles both in rats and man [8], [12], [13]. All these beneficial effects on bubble formation and DCS could be associated with antitadhesive and antithrombotic properties of NO and not to its vasodilator effect.

The results of our pilot study suggest that inhibitors of PDE-5 such as sildenafil are potentially dangerous pre-treatments, which can promote the occurrence of DCS. This promoting effect could be related to vasodilation with increased cerebral blood flow. Further studies, including the assessment of oxidative stress markers with production of peroxynitrites in CNS may be relevant to elucidate the underlying mechanisms of sildenafil in DCS.

In conclusion, since pre-treatment with sildenafil promotes the onset and severity of neurological decompression sickness, it is important to inform the community of divers from potential risks associated with inhibitors of PDE-5 treatment for diving activities; when considering their usage in the context of diving, careful discussion with a physician should be recommended.
